# Density of Key-Species Determines Efficiency of Macroalgae Detritus Uptake by Intertidal Benthic Communities

**DOI:** 10.1371/journal.pone.0158785

**Published:** 2016-07-14

**Authors:** Agnes M. L. Karlson, Clarisse Niemand, Candida Savage, Conrad A Pilditch

**Affiliations:** 1 Department of Environmental Science and Analytical Chemistry, Stockholm University, Stockholm, Sweden; 2 School of Science, University of Waikato, Hamilton, New Zealand; 3 Department of Marine Science, University of Otago, Dunedin, New Zealand; 4 Department of Biological Sciences, University of Cape Town, Cape Town, South Africa; The University of Sydney, AUSTRALIA

## Abstract

Accumulating evidence shows that increased biodiversity has a positive effect on ecosystem functioning, but the mechanisms that underpin this positive relationship are contentious. Complete extinctions of regional species pools are comparatively rare whereas compositional changes and reductions in abundance and biomass are common, although seldom the focus of biodiversity-ecosystem functioning studies. We use natural, small-scale patchiness in the density of two species of large bivalves with contrasting feeding modes (the suspension-feeding *Austrovenus stutchburyi* and deposit-feeding *Macomona liliana*) to examine their influence on the uptake of nitrogen from macroalgae detritus (i.e. measure of ecosystem function and food web efficiency) by other infauna in a 10-d laboratory isotope-tracer experiment. We predicted that densities of these key bivalve species and functional group diversity (calculated as Shannons H, a density-independent measure of community composition) of the intact infaunal community will be critical factors explaining variance in macroalgal per capita uptake rates by the community members and hence determine total uptake by the community. Results show that only two species, *M*. *liliana* and a large orbiniid polychaete (*Scoloplos cylindrifer*) dominated macroalgal nitrogen taken up by the whole community due to their large biomass. However, their densities were mostly not important or negatively influenced per capita uptake by other species. Instead, the density of a head-down deposit-feeder (the capitellid *Heteromastus filiformis*), scavengers (mainly nemertines and nereids) and species and functional group diversity, best explained per capita uptake rates in community members. Our results demonstrate the importance of species identity, density and large body size for ecosystem functioning and highlight the complex interactions underlying loss of ecological functions with declining biodiversity and compositional changes.

## Introduction

Considering the major community changes, including species losses, documented worldwide in recent years there is an urgent need to gain a mechanistic understanding of the relationship between biodiversity and ecosystem functioning—which ultimately affects the ecological services provided to humanity [1, 2]. Accumulating evidence shows that increased biodiversity has a positive effect on ecosystem functions, such as primary production, decomposition of organic matter and nutrient regeneration, but the pattern of response varies depending on the ecosystem and species investigated [1–4]. Much of what we know about the role of biodiversity in mediating ecosystem functioning stems from manipulative laboratory experiments. Although they have helped articulating hypotheses and provided mechanistic explanations for observed patterns they do not incorporate habitat complexity or allow long-term community dynamics and feedback processes to develop [5–7]. A key challenge in the field of biodiversity-ecosystem function research is to demonstrate whether the observed importance of biodiversity in controlled experimental assemblages also persists in natural systems [4, 8–10].

Biodiversity explains variation from the level of genes to ecosystems, with species richness (number of species) being the most commonly used measure in studies examining biodiversity and ecosystem function relationships. Species richness is representative of an environment as it is determined by prevailing biotic and abiotic conditions, as well as being a logistically achievable measure, and is therefore appropriate for such studies. Three main hypotheses have been proposed that relate the responses of ecosystem functioning to species richness. First, the linear or “rivet” hypothesis suggests that all species contribute critically and approximately equally to ecosystem function e.g. Lawton [11]. Second, the “redundancy” hypothesis suggests that ecosystems can lose many species with no consequences for ecosystem performance, as long as the major functional groups are still present, i.e. it is not the number of species per se which is important but the functional traits of the species [11, 12]. Redundant species are considered necessary only to ensure ecosystem resilience to perturbation [12]. Third, the “idiosyncratic” hypothesis states that species diversity affects ecosystem functioning, but not in a predictable direction, because the roles of individual species are complex and context-dependent [11, 13]. Biodiversity-ecosystem function relationships within any system may be determined by any combination of these three hypotheses. However, there are further important components of biodiversity that may affect these relationships, including the density of a species [14, 15]. In many cases it has been shown that certain key-species, rather than species richness, can have a disproportionate effect on ecosystem functioning such as nutrient cycling and productivity e.g. [16–19]. Loss of a key-species would result in a rapid decline in ecosystem functioning [20] (c.f. rivet hypothesis) as this species is unique and cannot be replaced by another species with similar functional traits (c.f. redundancy hypothesis). Changes in species abundance patterns may have important consequences for ecosystems long before a species is threatened by extinction [14]. At local scales, variations in the absolute density and relative abundance of species can modify biodiversity-ecosystem functioning relationships. For example the per capita performance of individual species may increase as their density declines, reflecting reduced intraspecific competition [21]. Also, decreased relative abundance of one species may likely alter complementary resource use or facilitation [22]. The hypotheses listed above would have difficulty in accounting for these common shifts in biodiversity.

Marine soft sediments cover more than 70% of Earth’s surface and play a critical role in the global storage and cycling of nutrients and energy [7, 23, 24]. Benthic invertebrate species often contribute idiosyncratically to ecosystem functioning with their impact strongly dependent on species identity and their functional role e.g. [25–27]. There is also some support for the redundancy hypothesis. Raffaelli et al. [28] grouped species by functional group according to their mode of bioturbation and found that increased species richness of benthic macrofauna belonging to different functional groups had a significant effect on nutrient fluxes from sediments, while increased species richness within the same functional group had no effect. Complete extinctions of regional species pools are however comparatively rare in the marine benthos whereas compositional changes and reductions in abundance and biomass are common [29, 30]. These changes in benthic abundance and biomass can be important drivers of ecosystem functioning as they direct species dominance patterns and functioning (e.g. infaunal community structure and diversity [31]; bioturbation potential and degradation patterns; [18, 32, 33]).

New Zealand sandflats provide an ideal system to investigate the contribution of species composition and abundance to ecosystem functioning because the macrofaunal community is species rich and has diverse functional groups. Using small-scale patchiness (0.01 m^2^) in the density of key-species, we compared the uptake of macroalgal detritus by the benthic infaunal community. This process is a fundamental ecosystem function where benthic infauna converts dead organic material to secondary production, which is available for higher trophic levels such as fish. The isotope tracing technique enables quantifiable measurement of detrital uptake by all species in the community; resolving trophic relationships and the outcomes of species interactions which amount to uptake at the community level [21]. This approach also enables the detection of subtle diversity effects, which could be masked from key-species effects when studying cumulative processes only (e.g. nutrient fluxes or bioturbation depth [28]). The use of intact cores with natural infaunal communities under controlled laboratory conditions and the ability to relate macroalgal uptake to the behaviour of individual species and their distribution in the sediment gives greater insight into the mechanisms underlying the relationships between biodiversity and ecosystem functioning than is typical of studies where the contribution of individual species to community interactions cannot be disentangled.

The two dominant bivalves on New Zealand intertidal sandflats, the large, mainly surface deposit-feeding deep-burrowing tellenid *Macomona liliana* and the large suspension-feeding endemic venerid *Austrovenus stutchburyi*, influence sediment characteristics and community composition, which affects ecosystem functions such as nutrient fluxes, metabolism and primary production [19, 32, 34]. We predict that densities of these large key-species will drive the patterns of uptake of algal detritus by macrofauna (c.f. the key-species hypothesis, a variant of the rivet hypothesis [20]) but that higher functional group diversity as measured by diversity indices will also contribute in explaining uptake (redundancy hypothesis). Our knowledge of the natural history of key-species allows us to hypothesise that i) higher densities of *M*. *liliana* will facilitate uptake of macroalgae detritus by sub-surface deposit feeders, since they draw organic material from the sediment surface with their inhalant siphon and defecate at depth, enhancing the concentration of organic matter at 5–10 cm below the sediment surface [35]. In contrast, ii) high densities of *M*. *liliana* should decrease uptake by small surface-feeding infauna due to exploitative and interference competition in the surface layer (as found for macrofauna-meiofauna interactions [36]). Furthermore, we hypothesise that iii) high densities of the clam *A*. *stutchburyi* will facilitate macroalgal uptake by surface-feeding infauna, since clams, if feeding on resuspended macroalgal detritus, would produce organic-rich deposits in the surface sediment thereby facilitating uptake by other infauna [37]. However, in laboratory conditions where resuspended material will settle again, their bioturbation and mixing in the upper centimetres of sediment [32, 38] may rework algal detritus into the sediment and eventually increase food access also for sub-surface feeders. To summarize, in this study we investigate whether relationships between species diversity, functional group diversity and densities of key-species and ecosystem functioning (detritus uptake) occur in natural communities. We test this using a multiple regression approach; uptake of algal detritus at both an individual level (per capita uptake by each species) and at the community level (total uptake by the whole community) is evaluated in relation to these measures of community structure.

## Methods

### Macroalgal labelling

The macroalgal species *Ulva* sp. which blooms in estuaries [39] and later decomposes in soft sediments was collected on Oct 10, 2011, from northern Tauranga Harbour, New Zealand, at low tide. Healthy looking thalli were rinsed in GFC filtered seawater and distributed among aquaria comprising an *Ulva* to seawater ratio of 10 g ww L^-1^. Two days later, we labelled *Ulva* with stable carbon and nitrogen isotopes by adding 5% Na^15^NO_3_, 10% (^15^NH_4_)_2_SO_4_ and 99% NaH^13^CO_3_ to the seawater in quantities similar to Rossi [40]. We also added KH_2_PO_4_ according to the Redfield ratio to improve growth condition and hence ensure that assimilation of isotopes would result in sufficient isotope enrichment. *Ulva* was placed in a constant temperature room set at 18°C under on a 12 h light:dark cycle for 6 d. The thalli were then carefully and repeatedly rinsed in MilliQ water, quickly dried using paper towels, freeze-dried and ground to a fine homogenised powder using a ball mill. The labelled macroalgae was sampled for stable isotope analyses (see below) and stored frozen until the start of the experiment. Isotope analyses confirmed a strong labelling of the *Ulva* material; δ^15^N = 9597 ± 95 ‰, δ^13^C = 1745 ± 11‰ compared to unlabelled *Ulva*; δ^15^N = 8 and δ^13^C = -12.

### Collection of intact cores

On 22^nd^ Nov 2011, we collected intact sediment cores from Tuapiro Point, Tauranga Harbour. Animal ethics approval/permits were not sought as benthic invertebrate fauna used in this study are exempt from the Animal Welfare Act 1999. The collection of benthic fauna was undertaken with a Ministry of Primary Industries Special Permit (560) Client Number 8770024. At low tide, 78 cores (12.5 cm in diameter, 20 cm deep) were selectively taken from a known *Austrovenus stutchburyi* bed (S 37° 29.390, E 175°57.014) and *Macomona liliana* bed (S 37° 29.344, E 175°57.094) located approx. 50 m apart. Sediment properties were similar at both sites; the median grain size was 183 and 192 μm, mud content (<63 μm) 5.2 and 3.3% and organic matter content 2.1 and 1.8% at the *Austrovenus* and *Macomona* sites, respectively.

Salinity and temperature was 29.3 and 16.7°C on the outgoing tide and 25.9 and 20.1°C on the incoming tide on the day of sampling. The distinct feeding tracks of *M*. *liliana* and the holes created by anemones (*Anthopleura aureoradiata)* attached to *A*. *stutchburyi* enabled estimates of their respective abundances so that cores from low to high bivalve density could be collected and to avoid destructive sampling of individuals close to core edges. Preliminary sampling indicated higher species richness at the *Austrovenus* than the *Macomona* site so the former site was sampled more intensively. After sacrificing some cores for initial analyses (see below) there were 41 and 29 experimental cores for the *Austrovenus* and *Macomona* site respectively to which labelled *Ulva* were added.

Back at the laboratory, cores from the two sites were randomly allocated to 12 tanks that were connected to a flow-through seawater system that generated a 12 h tidal cycle with a 6 h immersion/emersion period. The cores were fitted with an 800 μm mesh net around the circumference of the core that was extended above the simulated “high tide” mark to prevent amphipods escaping. An 800 μm mesh net also covered the base of each core so water could drain through the sediment with the rise and fall of the “tide”. The thermo-constant laboratory had windows, which allowed natural light to reach the cores (PAR 4.3±2 μE, 15 cm above the sediment surface). The light:dark cycle was 12:12 h (8 am: 8 pm). Artificial saltwater was used in the experiment (salinity 29.4) and temperature set at 19°C.

### Start of the experiment

The cores were left to acclimatize for two tidal cycles. At low tide the next day (23 Nov), 0.60 ± 0.01 g dw of the labelled, finely ground *Ulva* was mixed with 20 ml seawater and added to each core by carefully spreading it evenly on the sediment surface using a Pasteur pipette. Recovery of added *Ulva* from sub-sampling sediment in six cores containing few (< 2) *M*. *liliana* and *A*. *stutchburyi* after 24 h was 97± 3%, supporting visual observations (S1 Fig) and verifying that detrital recovery at the end of the experiment could be attributed to faunal activity rather than resuspension and loss due to the simulated tidal cycles.

### Experimental procedures and termination of experiment

The experiment was checked twice a day and occasionally dead *A*. *stutchburyi* were carefully removed from the surface sediment. After 10 d each core was sieved on a 500 μm mesh and fauna preserved in 70% ethanol until sorted to species level under a stereomicroscope. All specimens were counted and biomass measured (after drying at 60°C) or estimated. For larger polychaetes which were often incomplete, a width-biomass relationship (r^2^ = 0.84–0.92) was established from intact individuals of each species [41]. Bivalves were weighed without shells since we were interested in macroalgal uptake in organic material. The species abundance (core^-1^) and total biomass are given in S1 and S2 Tables.

The eleven most common macrofaunal species were selected for isotope analyses. Within species, similar sized individuals were selected to minimize biomass/growth dependent enrichment [42]. Only adult individuals were used for isotope analyses with the exception of *Naineris* sp. which were present as juveniles only. For abundant species with a small biomass (*Aonides trifada*, *Prinospio aucklandica*, *Naineris* sp.), the first 10–20 individuals encountered (to obtain enough biomass for analyses) from each core were collected and transferred to a pre-weighed tin capsule. For amphipods (*Parawaldeckia* sp.), about 6 individuals core^-1^ were used. Larger species (*Scoloplos cylindrifer*, *Orbinia papillosa*, *Nereis* sp., *Heteromastus filiformis*, *Nucula* sp. *M*. *liliana* and *A*. *stutchburyi*) were weighed or measured individually, pooled then homogenised to get a representative sample for isotope analyses from each core. Other species either had too small biomass for isotope analyses or did not occur in enough cores to allow statistical analysis, however a few of these additional species were screened for enrichment to improve community uptake estimates.

### Isotope analyses and calculations

Aliquots (about 2 mg dw) of samples for isotope analyses of carbon and nitrogen were packed in tin capsules and analysed at the Chemistry Department, University of Otago, in a Carlo Erba NA1500 elemental analyser coupled to a Europa 20/20 mass spectrometer. Internal standards, which were calibrated against international standards, were run in each batch of samples. The average standard deviation for all runs was ± 0.2 for δ^15^N and ± 0.1 for δ^13^C. The C and N isotope ratios are expressed in the ‰ notation, using the equation:

$$\delta (‰)=\left(\frac{R_{sample}}{R_{standard}}\text{-}1\right)\times 10^{3}$$ (1)

where R is the ratio between the heavy and light isotopes (^13^C:^12^C or ^15^N:^14^N). The stable isotope ratio, denoted by δ, is defined as the deviation in ‰ from an international reference standard (Vienna PeeDee Belemnite for C, and atmospheric nitrogen gas for N). Higher δ values indicate a higher proportion of the heavy isotope.

To quantify the macroalgal (*Ulva* sp.) nitrogen (N) taken up in faunal tissue, a linear two-source mixing model was used [21]:

$$f1+f2=1;\;f1=(\delta_{sample}-\delta_{source2})/(\delta_{source1}-\delta_{source2})$$ (2)

where f1 is the proportion of *Ulva* N in the animal sample and f2 is the proportion of N derived from the initial sediment. The amount (mg) of *Ulva*-N taken up in each animal was calculated from the mixing model (proportion N from *Ulva*) and the total N content (mg) in the animal. This amount was extrapolated to the number of individuals of this species found in this core. To obtain community uptake of *Ulva*-N the species-specific total uptake values (based on core-specific density) were summed. Uncorrected δ values were used in the mixing model, since species-specific differences in fractionation [43] and fat content [44] were negligible compared to the strong labelling. C uptake is not shown since δ^13^C and δ^15^N enrichment were highly correlated for all species, Pearson product moment correlation r ˃ 0.95 (δ^13^C, δ^15^N, C and N content (%) in benthic fauna can be found in S3 Table).

### Functional group categorisation and selection of species for statistical analyses of uptake

All species were included in a biological traits matrix containing 32 traits based on an organism's living position, sediment topographic features created, the direction of sediment particle movement, the degree of motility, feeding behaviour, body size, shape and hardness [45, 46]. Based on these traits, species were assigned to functional groups [47], Table 1. The large key-species (*M*. *liliana* and *A*. *stutchburyi*) separated into single species functional groups (deposit-feeding bivalves and suspension-feeding bivalves). The overall prediction was that densities of these two functional groups (species) as well as the functional group and species diversity of the community would determine per capita uptake by infauna, and hence total community uptake (summed uptake by all community members). Species diversity and functional group diversity were calculated for each core using Shannon’s H’ which accounts for both abundance and evenness of the species (or functional groups) present and is therefore a density-independent measure of community composition. In addition to key species density and diversity indices we also included the densities of another four functional groups as explanatory variables in statistical analyses since their biomass and/or abundance dominated community structure (i.e. they constituted 73 ± 13% and > 95% of total abundance and biomass respectively). The additional four functional groups selected were: “Head-down deposit feeders” (also represented by only one species, the capitellid *Heteromastus filiformis*), “Large, mobile deposit-feeding polychaetes” (mainly Orbiniids, dominated by *S*. *cylindrifer*), “Large, mobile predators/scavengers” (mainly Nereids and Nemertins) and “Small, surface-deposit-feeding polychaetes” (mainly spionids, highly abundant). See Table 1 for details on the classification of all functional groups and Table 2 for site macrofauna metadata.

**Table 1:** Species and functional group assignment. Each species was assigned to a functional group (FG) based on Greenfield et al.[47]. Densities of FGs in bold (1–6) were included as explanatory variables in statistical analyses. Only adult specimens of *S*. *cylindrifer*, *M*. *liliana* and *A*. *stuchburyi* were included in the FG, as juveniles were expected to confound possible density effects. For *Naineris* sp. only juveniles were found. Species in bold were selected for isotope analyses and used as response variables in separate statistical tests. Underlined species were screened for isotope enrichment (per capita uptake) but not included in statistical analyses because of insufficient enrichment (e.g. *A*. *stutchburyi*) or low abundance.

FG	Body	Feeding	Position	Movement	Species
1	**calcified**	**suspension**	**top 2 cm**	**freely mobile**	*Austrovenus stutchburyi*
2	**calcified**	**deposit**	**deep**	**limited mobility**	***Macomona liliana***
3	**soft**	**deposit**	**below surface**	**freely mobile**	***Orbinia papillosa; Scoloplos cylindrifer;*** *Scolecolepides benhami*
4	**soft**	**deposit**	**below surface**	**limited mobility**	***Aonides trifida; Prionospio aucklandica; Naineris* sp.;** *Magelona dakini*, *Scolelepis* sp; *Paradoneis lyra*,
5	**soft**	**deposit, head-down**	**below surface**	**limited mobility**	***Heteromastus filiformis***; *Heteromastus sp*.
6	**soft**	**predator/scavenger**	**below surface/deep**	**freely mobile**	***Nereididae*** (unspecified); *Phyllodocidae*; *Nemertean*
**7**	rigid	deposit/predator/scavenger/grazer	top 2 cm	freely mobile	***Parawaldeckia spp*.;** *Phoxocephalidae spp*
**8**	calcified	deposit	top 2 cm	limited mobility	***Nucula hartvigiana***
**9**	calcified	suspension	attached	not mobile	Barnacle (unspecified); Limpet (unspecified)
**10**	calcified	deposit/predator/scavenger/grazer	above surface	freely mobile	*Cominella glandiforme;Diloma subrostrata; Zeacumantus lutulentus*
**11**	soft	suspension	attached	not mobile	*Anthopleura aureoradiata*
**12**	soft	suspension	attached, tube	not mobile	*Boccardia syrtis*
**13**	soft	deposit	deep	freely mobile	*Capitella* sp.
**14**	soft	predator/scavenger	top 2 cm	limited mobility	*Edwardsia* sp.; *Oligochaeta*
**15**	rigid	predator/scavenger	above surface	freely mobile	*Halicarcinus* (unspecified, juvenile)
**16**	rigid	predator/scavenger	below surface, burrow	freely mobile	*Heterosquilla* sp.

**Table 2:** Macrofaunal metadata. Differences between the *Austrovenus* and *Macomona* sites in terms of infaunal species richness, functional group richness (FG), Shannon diversity index forspecies (H’SP) and functional groups (H’FG), total density of individuals and the density of the key FG, (see Table 1 for explanations to abbreviations). Values are mean ± 1 SD. Headings in bold are predictors for statistical analyses.

	*Austrovenus* site	*Macomona* site
Species richness (# core^-1^)	15.9 ±2.4	8.9 ± 1.5
FG richness (# core^-1^)	9.9 ±1.3	5.9 ±1.2
**H' SP** (core^-1^)	2.2 ± 0.2	1.8 ±0.2
**H' FG** (core^-1^)	1.7 ± 0.2	1.2 ±0.2
Density (ind. core^-1^)	125±46	49±16
**FG1** (ind. core^-1^)	7.0 ± 6.1	0.5 ±0.7
**FG2** (ind. core^-1^)	3.6±2.2	3.6±2.3
**FG3** (ind. core^-1^)	14.6±7.3	6.3± 5.9
**FG4** (ind. core^-1^)	40.5±24.8	20.7±9.3
**FG5** (ind. core^-1^)	11.0±8.6	0
**FG6** (ind. core^-1^)	10.9±6.3	5.0±2.6

As response variables in the statistical approach taken (described under Data analyses and statistics), we used δ^15^N enrichment of the ten most abundant species (Table 1, one test for each species), and total uptake of *Ulva*-derived nitrogen by the macrofaunal community. Only three of the selected species were abundant at both sites (see results) and so statistical analyses were restricted to within-site comparisons with the exception of community uptake. Differences in isotope enrichment among species depends partly on differences in feeding mode [21, 48] and partly on differences in growth rate and metabolic turnover, resulting in differences in time to reach isotopic equilibrium with the diet [42]. For this reason, we avoided statistical comparisons in δ^15^N enrichment among species (for simplicity, referred to as per capita uptake throughout the paper). Since *A*. *stuchburyi* did not show any per capita uptake it was excluded as a response variable (but still included as a predictor variable, see above).

### Data analyses and statistics

#### Differences in macrofaunal community composition, biomass and total macroalgal N uptake between sites

Multidimensional scaling using principal coordinate analysis (PCO) and permutational ANOVA (Permanova) as implemented in PERMANOVA+ of PRIMER v6 [49] were used to assess inter-site differences in community biomass and species and functional group composition. Analyses were based on the Bray-Curtis Similarity Index and fourth-root transformed abundance data [49]. For PCO analyses we considered species/functional groups with a Spearman correlation > 0.6 with any of the first two ordination axes as significantly contributing to the difference between sites. Community biomass and community uptake of *Ulva*-derived N calculated for each core (see methods) was tested for differences between sites using Permanova. Biomass normalised N uptake (core-specific total uptake divided by total biomass of the core using only those species contributing to uptake (i.e. excluding *A*. *stutchburyi* biomass)) was tested in the same way to account for inter-site differences in biomass.

#### Predictors of community macroalgal N uptake

To test the overall prediction that community macroalgal N uptake was determined by density of functional groups and the functional diversity of the community, the relationship between community macroalgal N uptake and selected explanatory variables (that included total community biomass (all species) and those listed in Table 2) was assessed for each site separately, using distance-based linear models (DistLM) in PERMANOVA+ of PRIMER v6 [49]. DistLM is a multiple regression routine where a resemblance matrix (in this case based on Bray-Curtis distance of community macroalgal N uptake values using cores as samples) is regressed against a set of explanatory variables. Prior to analyses both response data and explanatory variables were square-root transformed to conform to normality. Skewness of the explanatory variables was inspected using pair-wise Draftman plots of all variable combinations. The explanatory variables were generally not strongly correlated to each other (Pearson’s r < the critical 0.95 according to [49]) and distributions were not strongly skewed. See Table 3 for relationships between the explanatory variables at each site and for sites combined. Marginal DistLM was first used to determine which variables accounted for a significant proportion of N uptake when considered alone in the model, ignoring all other variables. The variables included in the final DistLM-models for each species and site were selected using the ‘best’ selection procedure, which utilizes all possible combinations of explanatory variables to determine which combination accounts for the greatest proportion of uptake explained in the models R^2^ based on the corrected Akaike information criterion (AICc). To remove the effect of differential biomass between sites, biomass-normalized community uptake from both sites was tested in a DistLM with the addition of a categorical factor, site (*Macomona* or *Austrovenus*).

**Table 3:** Correlations between the predictors used in statistical analyses. Spearman rank correlations (ρ) between explanatory variables (densities of functional groups and diversity indices) used in DistLM analyses (Tables 4, 5 and 6). Values in bold are significant at p < 0.05. (A) *Austrovenus* site, (B) *Macomona* site and (C) both sites pooled. Abbreviations are defined in Tables 1 and 2. na = non-applicable predictor (this FG was missing for this site).

**(A)**	**H’SP**	**H’FG**	**FG1**	**FG2**	**FG3**	**FG4**	**FG5**
**H’FG**	**0.63**	** **	** **	** **	** **	** **	** **
**FG1**	**0.32**	**0.61**	** **	** **	** **	** **	** **
**FG2**	0.16	-0.06	-0.09	** **	** **	** **	** **
**FG3**	-0.05	0.29	0.07	-0.17	** **	** **	
**FG4**	-0.3	**-0.59**	-0.09	-0.08	-0.14	** **	
**FG5**	0.24	**0.49**	**0.56**	-0.27	0	0.07	
**FG6**	**0.32**	**0.34**	0.21	**-0.39**	0.1	-0.12	**0.43**
**(B)**	** **	** **	** **	** **	** **	** **	** **
**H’FG**	**0.88**	** **		** **		** **	
**FG1**	0.19	0.34					
**FG2**	0.33	**0.44**	0.01				
**FG3**	0.25	0	-0.26	0.31			
**FG4**	**-0.57**	**-0.64**	-0.19	-0.09	0.03		
**FG5**	na	na	na	na	na	na	
**FG6**	-0.07	-0.11	-0.07	-0.23	-0.04	0.19	na
**(C)**							
**H’FG**	**0.84**		** **		** **		** **
**FG1**	**0.68**	**0.79**			** **	** **	** **
**FG2**	0.16	0.08	-0.04				
**FG3**	**0.4**	**0.48**	**0.4**	0			
**FG4**	0.12	0	**0.32**	-0.11	0.21		
**FG5**	0.24	**0.49**	**0.56**	-0.27	0	0.07	
**FG6**	**0.49**	**0.53**	**0.46**	**-0.25**	**0.3**	**0.25**	**0.43**

**Table 4:** Predictors of total community uptake of *Ulva*-derived nitrogen. DistLM marginal test results reporting the proportion of total community N uptake at the *Austrovenus* (n = 41) and *Macomona* (n = 29) sites and both sites combined (biomass normalized) explained by diversity indices and FG densities (see Tables 1 and 2 for abbreviation definitions). Marginal tests results describe how much variation each variable explains when considered alone, ignoring other variables. The (+) or (-) sign denote direction of the relationship, na = non-applicable predictor. Significant relationships are shown in Fig 4.

Explanatory variable	*Austrovenus* site	*Macomona* site	Both sites
Total biomass		na^1^	na^2^
**H’SP**	0.08 (-)*	0.10 (+)*	
**H’FG**		0.13 (+)**	
**FG1**			0.20 (+)***
**FG2**	0.17 (-)***	0.58 (+)***	0.43 (-)***
**FG3**	0.44 (+)***	0.17 (+)**	0.22 (+)***
**FG4**			0.04*
**FG5**		na^3^	0.30 (+)***
**FG6**			
**Site**	na	na	0.24***

*p < 0.1

**p < 0.05

***p < 0.01

na^1^ total biomass and *Ml* density were highly correlated (ρ > 0.95), thus, total biomass was not included in the analyses

na^2^ uptake was normalized for biomass when combining both data from both sites

na^3^ FG3 was absent from this site

**Table 5:** ‘Best’ model of total community uptake of *Ulva*-derived nitrogen. Results from the ‘best’ model selection procedure for different numbers of predictor variables at the *Austrovenus* site, *Macomona* site and both sites pooled (biomass normalized). AICc denote corrected Akaike information criterion and R^2^ is the total cumulative variance explained by the model. See Tables 1 and 2 for definitions of other abbreviations.

Number of variables	AICc	R^2^	Predictor variables
***Austrovenus* site**			
1	227.86	0.44	FG3
2	224.18	0.52	FG3, FG2
3	219.73	0.59	FG3, FG2, H’SP
4	220.60	0.61	FG3, FG2, H’SP, FG1
5	221.22	0.63	FG3, FG2 H’SP, FG1, FG6
***Macomona* site**			
1	155.17	0.58	FG2
2	151.35	0.66	FG2, FG3
3	152.09	0.69	FG2, FG3, FG1
4	153.11	0.71	FG2, FG3, FG1, FG4
5	155.06	0.72	FG2, FG3, FG1, FG4, H’FG
**Both sites**			
1	417.68	0.43	FG2
2	397.16	0.55	FG2, site
3	395.18	0.61	FG2, site, FG3
4	393.65	0.63	FG2, FG5, H’SP, site
5	392.28	0.65	FG2, FG3, FG5, H’SP, site

**Table 6:** Predictors of per capita uptake (δ^15^N enrichment). A summary of marginal test and ‘best’ model results for (A) *Austrovenus* site and (B) *Macomona* site for the per capita uptake by each species (rows) as explained by species and functional group (FG) density and diversity indices (see Tables 1 and 2 for definitions of abbreviations). Numbers are the proportion of variance explained by single predictors (marginal tests) and the (+) or (-) denote direction of significant relationships. Values in bold denote parameters selected by AIC_C_ to be included in the ‘best model’ and the R^2^ is the total cumulative variance explained by the ‘best model’. Non-significant variables or variables not included in the ‘best model’ are not shown. na = non-applicable predictor. Significant relationships are shown in Fig 5.

Variable	H’ SP	H’ FG	FG1	FG2	FG3	FG4	FG5	FG6	R^2^
**a) *Austrovenus* site**									
*P*. *aucklandica*; n = 26		0.32; (+)**	0.12; (+)*	0.22; (-)**	0.10*	0.31; (-)***	0.20; (+)**	0.21; (+)**	0.51
*A*. *trifada*; n = 11		**>0.01**				**0.18**			0.39
*Parawaldeckia* sp.; n = 24				0.32; (-)***	0.13*		0.14; (+)*		0.35
*Nucula* **sp.;** n = 18	0.48; (+)***	0.31; (+)***		**0.02**			0.26; (+)***		0.68
*M*. *liliana;* n = 3**1**			**0.07**				**0.06**	**0.04**	0.17
***S*. *cylindrifer*; n = 26**							**0.05**	**0.03**	0.12
*O*. *papillosa*; n = 23	**0.10; (+)**	**0.01**	**0.04**						0.31
*Nereis* sp.; n = 23							0.13; (+)*	0.22; (+)**	0.29
*H*. *filiformis*; n = 35	0.09*	0.29; (+)***	0.17; (+)**	0.19; (+)***			0.35; (+)***	0.23; (+)***	0.54
**b) *Macomona* site**									
*Naineris* sp.; n = 25	**0.02**	**0.03**					na		0.14
*M*. *liliana*; n = 26			0.15; (+)**	**0.01**			na		0.17
*S*. *cylindrifer*; n = 21	**0.01**					0.22 (+)**	na	**0.04**	0.38

*p < 0.1

**p < 0.05

***p < 0.01

#### Predictors of per capita uptake (individual δ15N enrichment)

The association between δ^15^N isotope enrichment (per capita uptake) and the selected explanatory variables was assessed for each species and site separately using DistLM, as described above. This resulted in 12 species-specific models; nine for the *Austrovenus* site and three for the *Macomona* site. Since the main purpose of these individual uptake models was to generalize among responses and predictors we present only significant marginal results and the variables included in the ‘best’ model based on AICc. For those models where AICc values were within 2 units, the model with highest explanatory power was chosen rather than the most parsimonious model, since the purpose was to find the combinations of species that would best explain enrichment patterns. The specific hypotheses related to the effects of key species feeding mode on macroalgal N uptake by surface- and subsurface feeding infauna were determined by comparing whether *A*. *stutchbury* (FG1) or M. *liliana* (FG2) were included in the best model for a species with those particular feeding modes (Table 6).

## Results

### Community composition and sediment characteristics at the *Macomona* and *Austrovenus* sites

**Fig 1:**
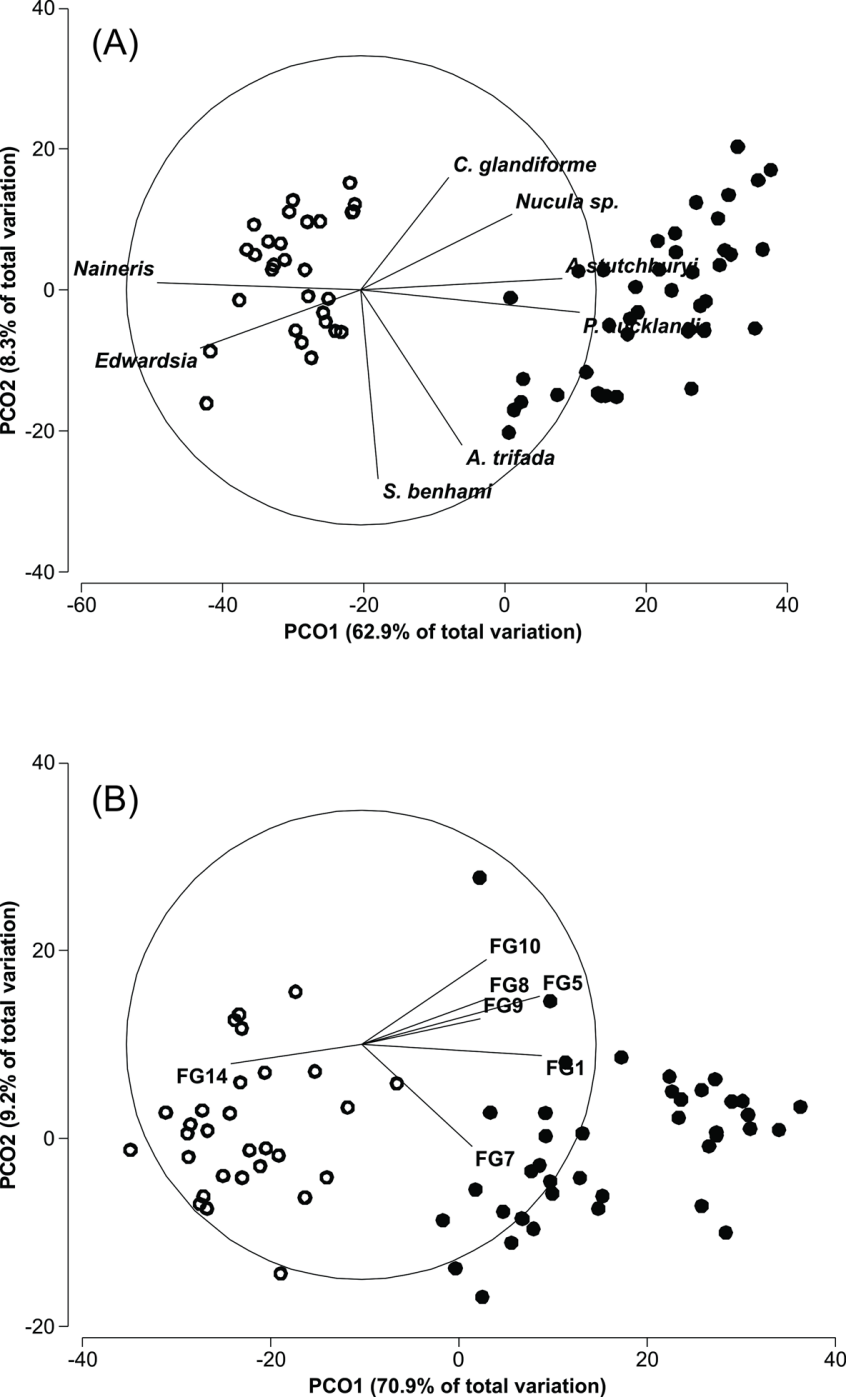
**Results of a PCO analysis of (A) species composition and (B) functional group composition.** Empty symbols represent the *Macomona* site and filled symbols the *Austrovenus* site. Only species or functional groups with a Spearman correlation > 0.6 are shown. To improve clarity, *A*. *aeuroradiata* and *Phoxocephalidae* were removed from (A) since they are highly correlated and nearly identical to the distribution of *A*. *stutchburyi*. Similarly, the distribution of *O*. *papillosa* was identical to *P*. *aucklandica* and Oligochaetes were identical to *Naineris* sp. See text for Permanova results and Table 1 for species and functional group explanations.

At the *Austrovenus* site 30 macrofaunal species and 13 functional groups were encountered, while at the *Macomona* site only 22 species and 9 functional groups were encountered (Table 2). There was a significant difference in macrofaunal community composition (based on species abundance) between the *Macomona* and *Austrovenus* sites (Permanova, Pseudo-F_1,67_ = 63.28, p = 0.0001, Fig 1A, S1 Table). The same clear separation between sites was obtained when functional group composition was used (Permanova Pseudo-F_1,67_ = 64.54, p = 0.0001, Fig 1B, Table 2). However, the species which dominated the biomass (*A*. *stutchburyi* and *M*. *liliana* and the orbiniid *Scoloplos cylindrifer*) were present at both sites. Other common infaunal species and taxa commonly occurring at both sites were the polychaetes *Nereis* sp., *Prinospio aucklandia*, *Scolecolepides benhami*, *Scolelepes* sp., *Nanieris* sp., the amphipod *Parawaldeckia* sp., the anemone *Edwardsia* sp. and Nemertines and Oligochaetes.

### Isotope enrichment of *Ulva* and infauna

**Fig 2:**
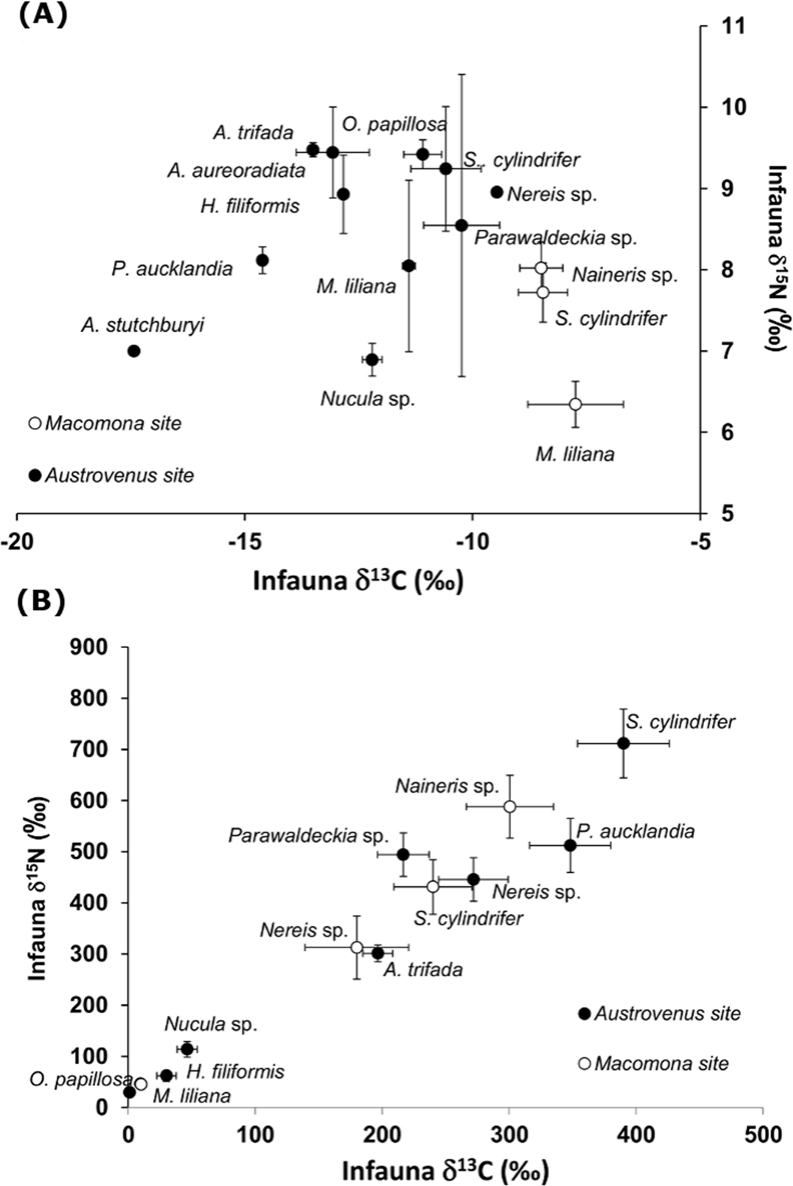
**Initial natural abundance isotope values (A) and final enriched isotope values for infauna after the addition of isotope enriched *Ulva* detritus (B).** The initial isotope values (mean ± 1 SD, n = 2–6) are shown for common species at both sites. The final values include only those species selected for statistical analysis (see methods) and the data represent the mean ± 1 SE (n = 23–35 except for *A*. *trifada* where n = 11 and *Nucula* sp. where n = 18).

The *Ulva* was highly enriched (δ^15^N = 9597 ± 95 ‰, δ^13^C = 1745 ± 11‰, mean ± SD, n = 3) relative to the sediment (c. δ^15^N = 6 ‰, δ^13^C = -15 ‰) and initial values for fauna (Fig 2A), enabling quantification of macroalgal uptake by benthic infauna (section below). Initial isotope values differed among species (Fig 2A, S3 Table). The isotope enrichment of the species at the end of the experiment (per capita uptake) varied both among and within species (Fig 2B, S4 Table). All species selected for statistical analyses were highly enriched compared to initial values, although in a few individuals (˂ 5%) of *M*. *liliana*, *O*. *papillosa* and *H*. *filiformis* minimal enrichment occurred. Anemones (*Anthopleura aureoradiata*) attached to *A*. *stutchburyi* were not expected to feed on *Ulva* detritus however the samples analysed for screening purposes revealed substantial enrichment (δ^15^N = 56 ± 30, δ^13^C = -5 ± 2, mean ± 1 SD, n = 6).

### Community uptake of macroalgal N in relation to predictors

**Fig 3:**
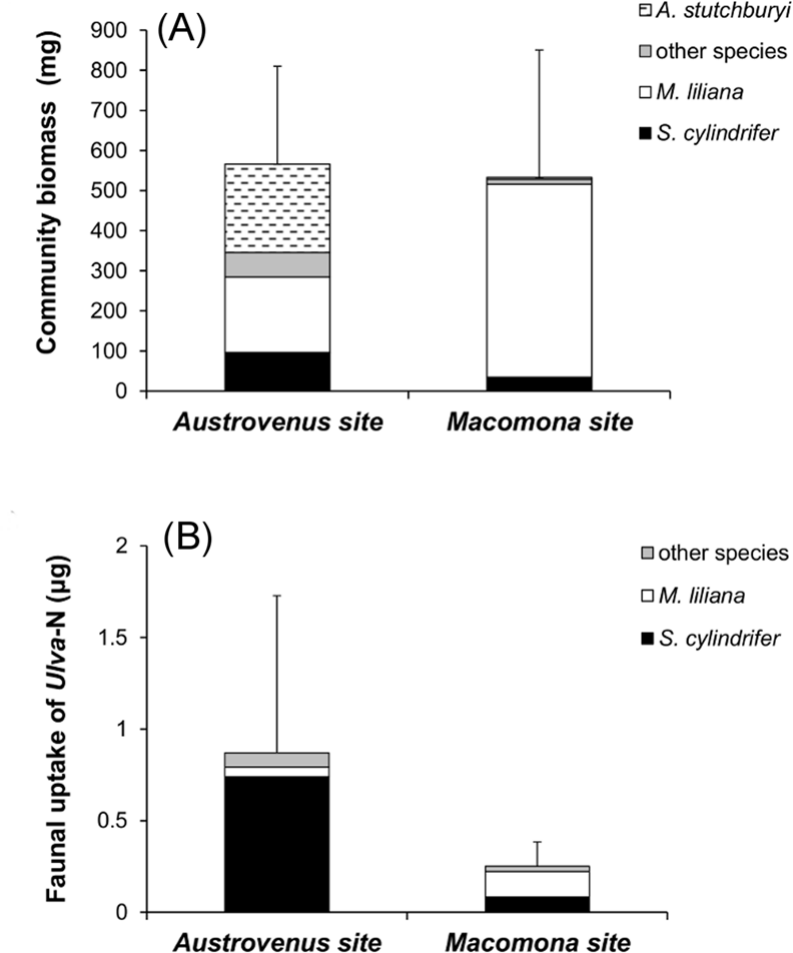
Infaunal community biomass and uptake of *Ulva*-derived nitrogen. Community biomass (shell-free dry weight) (A) and community uptake of macroalgal nitrogen (B) with the species contributing most at each site shown. Values are mean ± 1 SD.

Community infaunal biomass was similar between sites, 0.56 ± 0.25 (*Austrovenus* site) and 0.53 ± 0.32 mg core^-1^ (*Macomona* site) (Fig 3A), however the macrofaunal community at the *Austrovenus* sites had taken up approximately three times more *Ulva-*N than at the *Macomona* site (0.83 ± 0.86 vs 0.25 ± 0.13 mg; Permanova Pseudo-F = 16.779, p = 0.0001). This difference was even more pronounced (5 times) after normalizing uptake by the enriched biomass since *A*. *stutchburyi* did not contribute to uptake (Pseudo-F = 21.877, p = 0.0001). Two species, *M*. *liliana* and *S*. *cylindrifer*, were mainly responsible for the amount of *Ulva*-derived N taken up in faunal biomass during the experiment (Fig 3B). *S*. *cylindrifer* took up on average 89% of this nitrogen at the *Austrovenus* site and 33% at the *Macomona* site, whereas *M*. *liliana* took up 6% and 55% at the respective sites. This can be compared with the average contribution to community biomass by the same species; at the *Austrovenus* site, *A*. *stutchburyi*, *M*. *liliana*, and *S*. *cylindrifer* constituted 39%, 33% and 17% respectively; and at the *Macomona* site, 7%, 90% and 1%, respectively (Fig 3A).

**Fig 4:**
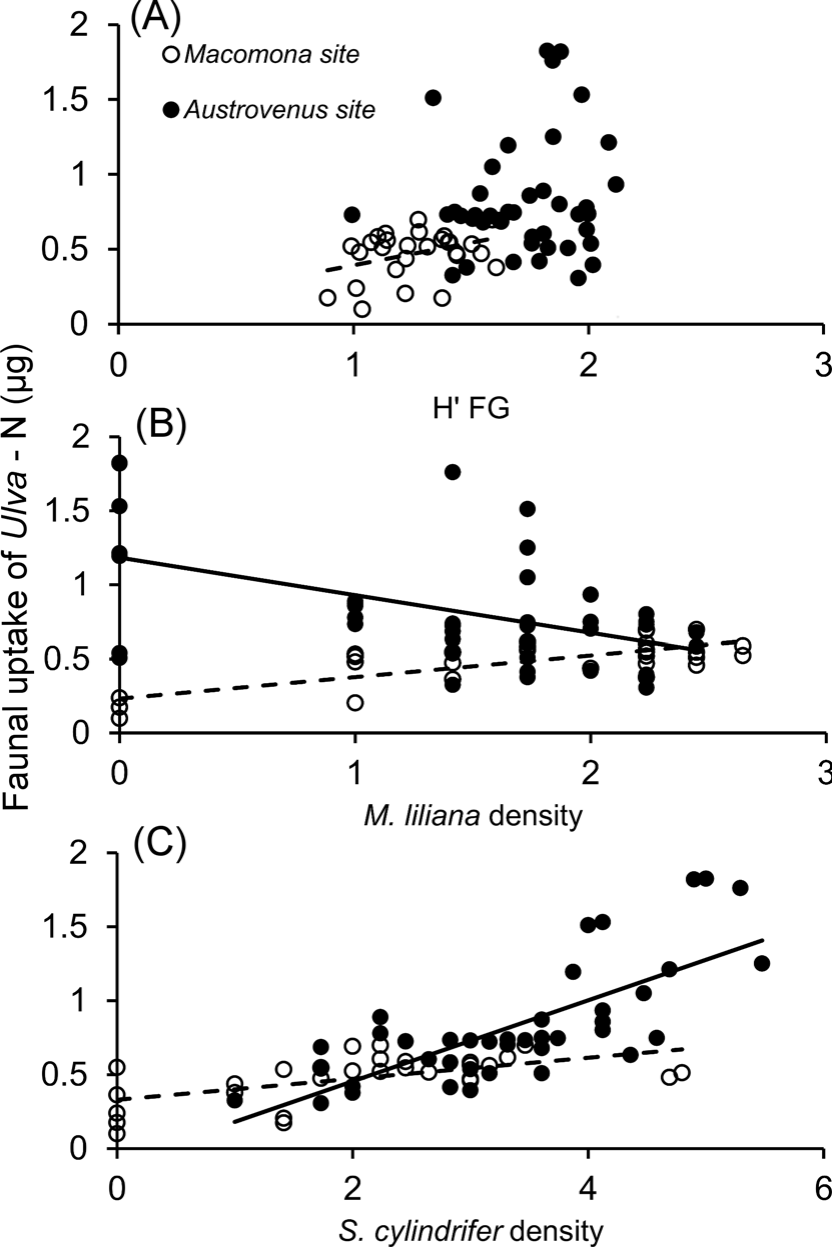
**Total community uptake of *Ulva*-derived nitrogen in relation to a) Shannon’s H’FG, and b) *M*. *liliana* density and c) *S*. *cylindrifer* density.** Empty symbols and dotted line represent the *Macomona* site and filled symbols and solid line the *Austrovenus* site. Both uptake and densities are square-root transformed. Only these relationships were significant (p<0.05) according to marginal tests in DistLM. See Table 4 for details on the statistical models.

Of the other species, only *Naineris* sp. (*Macomona* site, 7%) and *Nereis* sp. (*Austrovenus* site, 3%) contributed more than 1% to total community uptake. Accordingly, marginal tests showed that FG3, (i.e. *S*. *cylindrifer*) explained most of the variance in total community uptake at the *Austrovenus* site whereas *M*. *liliana* explained most of the variance at the *Macomona* site (Tables 3 and 4, Fig 4). Using biomass-normalized data across sites, the same two species as well as head-down feeders (i.e. *H*. *filiformis*), and site explained most of the variance. When the biomass effect of *M*. *liliana* is removed, the combined sites analysis shows that it has a negative effect on community uptake (in agreement with per capita uptake which is biomass independent). Even though not ranked as the most important predictors, it is worth noting that both functional group diversity (as predicted) and species diversity were significant in marginal tests or included in the ‘best’ model (Tables 4 and 5, Fig 4).

### Per capita uptake in relation to predictors

**Fig 5:**
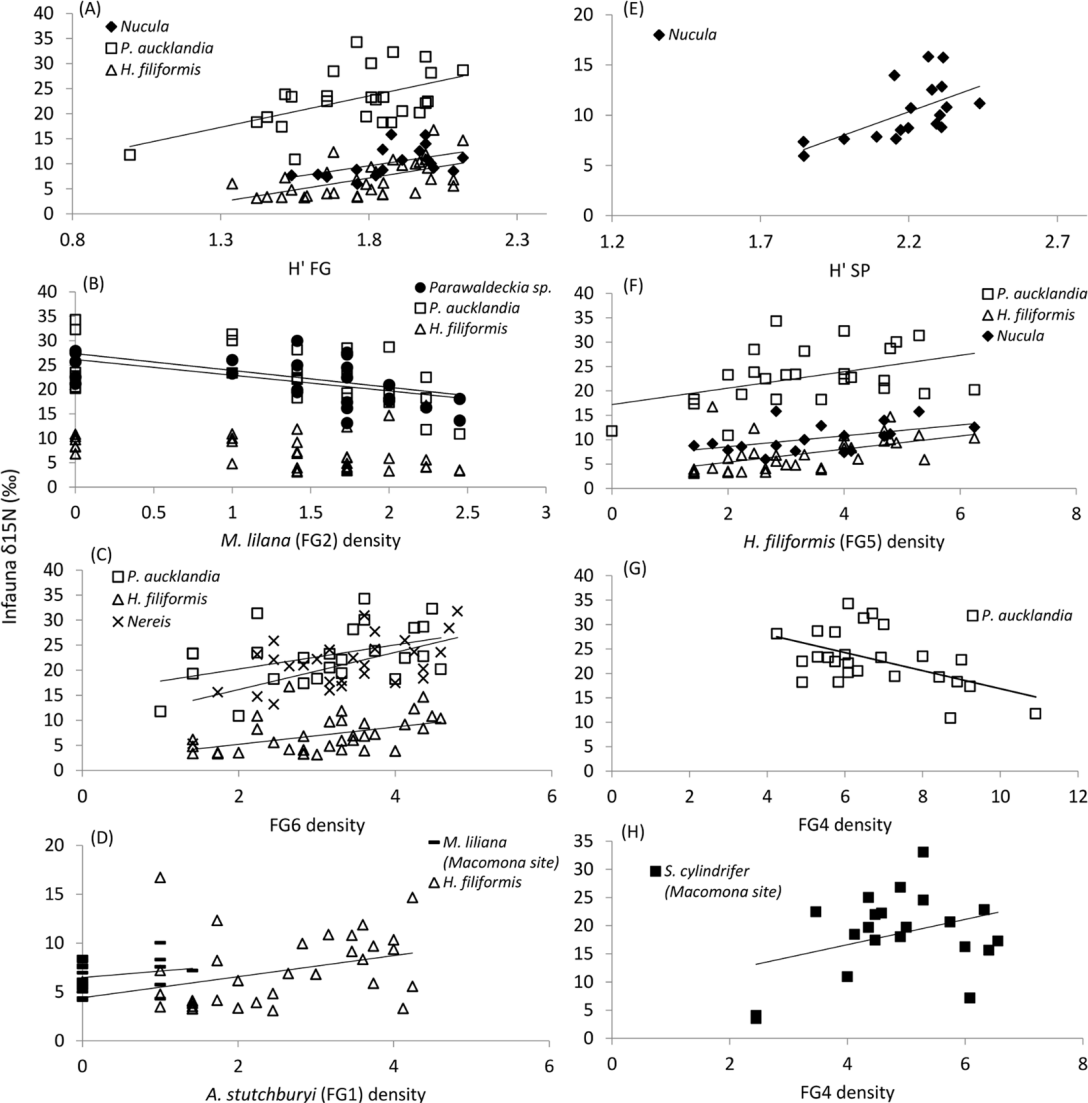
Per capita uptake (δ^15^N enrichment in individual species) in relation to species and functional group (FG) density and diversity indices. Species are represented by different symbols. All species are from the *Austrovenus* site except for *M*. *liliana* and *S*. *cylindrifer* in the bottom panels. Both uptake and densities are square-root transformed. Only significant relationships (p<0.05) according to marginal tests in DistLM are shown. See Table 6 for details on the statistical models and Tables 1 and 2 for definitions of abbreviations.

The results from site and species specific DistLM analyses of per capita uptake (δ^15^N enrichment on species level) are summarised in Table 6 (both marginal tests and ‘best’ models) and significant relationships are shown in Fig 5. At the *Austrovenus* site, *M*. *liliana* had, as hypothesised, a negative effect on the per capita uptake of the two surface feeders (*P*. *aucklandica* and *Parawaldeckia* sp.) and a positive effect on *H*. *filiformis* (Table 6). *H*. *filiformis* in turn, was positively associated with per capita uptake in species representing different feeding modes (Fig 5). As predicted, this was the case also for species and functional group diversity, which were positively correlated with per capita uptake in one and three species, respectively.

Per capita uptake of larger species (S. *cylindrifer*, *M*. *liliana*) had a lower proportion of variance explained than smaller species (this was true for both sites). *Naineris* sp. (abundant only at the *Macomona* site) had also no clear relationship to any of the explanatory variables included in the analyses.

## Discussion

This study shows that densities of only a few species in natural communities strongly influence the community uptake of macroalgal detritus. Using isotopically labelled macroalgae, we were able to relate the macroalgae detrital uptake to the ecological role of individual species and demonstrate the importance of densities of key-species for influencing ecosystem functioning. Using natural communities restricts us to a correlative statistical approach which cannot be confused with the species substitution approach commonly used in traditional biodiversity-ecosystem functioning studies. Still, the use of an isotope tracer provides greater insight into the mechanisms underlying the relationships between biodiversity and ecosystem functioning than is typical of studies where cumulative processes such as nutrient fluxes are the only endpoints measured e.g. [28]. Further, by using intact benthic communities with known functional traits, complex direct and indirect interactions among naturally co-occurring species could be discerned.

Although the core constrains mobility of the species, those selected a priori as key-species are sedentary and likely to be less affected. Similarly, most of the species analysed for isotope enrichment are small in body size and the cores could be thought of as mesocosms rather than microcosms. The only exceptions are the few large and mobile polychaete species encountered and the uptake for these species accordingly had poorer statistical models in terms of proportion variance explained. It is however possible that their uptake rates are more influenced by environmental conditions rather than community structure in the field due to their mobility. By sampling gradients in density of a priori selected key-species and measuring detrital uptake (the first step in benthic secondary production), our study bridges a gap between controlled experiments with selected species combinations and field data, where environmental conditions are difficult to control.

Uptake of macroalgal (*Ulva*) nitrogen by the whole community was three-fold (or five-fold when normalized for biomass) greater in the *Austrovenus* dominated site compared to the *Macomona* site. Previous studies have documented the importance of *Austrovenus stutchburyi* for ecosystem functioning due to physical properties of the bivalve bed and biological activities such as elevating sediment organic content through biodeposition [19, 32]. Our study demonstrates that *A*. *stutchburyi* also indirectly facilitates detrital uptake and food web efficiency in benthic infaunal communities, since its density was positively associated with higher functional diversity, species diversity and higher densities of the head-down feeder *Heteromastus filiformis* (FG5, Table 3) which, in turn, were all positively correlated to higher isotope enrichment on the individual level (hereafter referred to as per capita uptake of the *Ulva* nitrogen) for several species (Table 6). Further, *A*. *stutchburyi* density was positively related to per capita uptake in four species and it was the variable contributing most in explaining per capita uptake by *M*. *liliana* (at both sites). Although *A*. *stutchburyi* is a suspension-feeder, we expected some of the detritus, which was added as a fine powder to the sediment surface, to be resuspended from bioturbation activities and thereafter consumed and assimilated by the clam [50]. This was however not the case during the experimental period, perhaps due to their slow growth and metabolic turnover of bivalve foot muscle tissue (up to 1 year to reach isotopic equilibrium, [51]) but perhaps also due to the sheltered hydrodynamic condition in the experimental set-up minimizing resuspension processes, meaning that our results potentially underestimate its direct contribution to community uptake. Below we discuss mechanistic reasons for higher community uptake in the *Austrovenus* site compared to the *Macomona* site by examining the species level data.

Higher densities of the head-down feeder *H*. *filiformis*, which was absent from the *Macomona* site, was positively related to per capita uptake in three of the surface-dwelling deposit-feeders (the spionid *Prinospio aucklandica*, the amphipod *Parawaldeckia* sp. and the small bivalve *Nucula* sp.) as well as in the omnivorous *Nereis* sp. and *H*. *filiformis* itself at the *Austrovenus* site. It was also included as a predictor of macroalgal uptake in the best model for five species (Table 6). Possibly, buried *Ulva* detritus was brought to surface layers again through the feeding mode of this species. Similar positive interactions between head-down feeders and other species performance have been found, e.g. Weinberg and Whitlatch [52] reported increased growth of small suspension-feeding bivalves when kept in close proximity to a polychaete with this feeding-mode. The other small polychaete *Aonides trifada* was only abundant when *H*. *filiformis* densities were low so this particular relationship could not be properly tested here. It is however possible that *A*. *trifada* also feeds deeper in the sediment and should not be categorised as a surface dwelling deposit-feeder since these two species had very similar initial isotope signatures (Fig 2B); relatively depleted δ^13^C while enriched δ^15^N values, indicating feeding primarily on aged organic matter in the sediment [43, 53]. In support of this, per capita uptake by *A*. *trifada* was not influenced negatively by *M*. *liliana* which feeds mainly in the surface sediment. In other systems, deposit-feeders separate resources by depth in sediment and/or by feeding on different fractions of the organic matter e.g. fresh and aged [54, 55, 36]. Such niche differentiation increases resource utilization and thus promotes a positive biodiversity–ecosystem functioning relationship, as suggested by Karlson et al. [48]. The initial isotope values of fauna suggest that there is a broader range of primary producers supporting the food web at the more species rich *Austrovenus* site compared to the *Macomona* site. Although the aim of this study was not to disentangle the importance of different primary producers to the diet of macrofauna, the more depleted δ^13^C of *A*. *stutchburyi* indicates phytoplankton and macroalgae are the primary food sources whereas the enriched δ^13^C of *M*. *liliana* (at both sites) suggests feeding on microphytobenthos and seagrass detritus [50]. The generally more enriched δ^15^N values at the *Austrovenus* site compared to the *Macomona* site could indicate larger microbial conditioning of detritus that enrich nitrogen isotope values [43], perhaps also an effect of the higher density of individuals and higher species richness at this site. Interpretation of these differences, however, requires caution since the fauna were preserved in ethanol prior to analyses which may enrich δ^13^C values by a few ‰ [56] although other studies have found negligible effects from ethanol preservation on δ^13^C or δ^15^N e.g. [57].

In contrast to the positive effect of the head-down feeding *H*. *filiformis*, as hypothesised, higher densities of *M*. *liliana* were negatively associated with per capita uptake in two surface-feeding species; *P*. *aucklandica* and the amphipod *Parawaldeckia* sp. (both in marginal tests and in the best model results). This is likely due to the removal of added detritus from the surface sediment to deeper layers by the bivalve, and partly through consumption and defecation (as evident from the enriched isotope signal in *M*. *liliana* tissues demonstrating uptake of *Ulva-*derived nitrogen). There is a similar situation in the species-poor Baltic Sea, where the functionally and morphologically similar deposit-feeding bivalve *Macoma balthica* reduces access to food for other surface-feeding species, including amphipods [58, 48] and through interference competition lowers uptake rates of phytodetritus by meiofauna [36]. An alternative explanation is that increased oxygenation from the feeding mode of bivalves results in rapid mineralization of the organic matter by the bacterial community [21]. *M*. *liliana* generates pore-water pressure gradients during their feeding and burrowing behaviour that may stimulate bacterial activity through alteration of sediment oxygen dynamics [35]. The hypothesised increase in macroalgal uptake by sub-surface feeders, i.e. *H*. *filiformis*, along with higher densities of *M*. *lilana* (defecating at depth) was partly supported by our data (Table 6). Even more important in predicting *H*. *filiformis* per capita uptake was however higher functional group diversity (as Shannon H’FG), suggesting that more of the added material reached deeper in the sediment when more bioturbation modes are present. In a modelling study, Solan et al. [59] found that loss of species richness leads to a decline in bioturbation depth.

Larger species, e.g. *M*. *liliana*, *S*. *cylindrifer* and *Nereis* sp. had generally lower proportion of their respective per capita uptake explained by densities of other species/functional groups. For polychaetes, this is most likely due to their mobility, which enables them to feed in the whole sediment column. Interestingly, the functional group of large scavengers were selected in the best model for these species as well as for *H*. *filiformis*. We speculate that pre-conditioning of the refractory macroalgal food source resulting from feeding activities by e.g. *Nereis* sp., which is an opportunistic omnivore (the first species to show high uptake of isotopically labelled *Ulva* in the field after only 1 d of incubation), will facilitate uptake for the other species. This pre-conditioning is not likely to influence the isotope signal of the *Ulva* food source, since isotope fractionation effects are negligible compared to the strong enrichment from the labelled macroalgae, especially in a 10 d experiment. Bioturbation activities by *Nereis* sp. result in spatially redistributed food sources, improving its availability to bacteria and hence promoting stable co-existence through such scale-based partitioning of resources [60].

Species diversity (as Shannon H’SP) was positively associated with isotope enrichment in only one species, *Nucula* sp. (both in marginal tests and selected in the best model) while functional group diversity was significant for three species (*Nucula* sp., *P*. *aucklandica*, *H*. *filiformis*), although only selected in the best model for *H*. *filiformis*. Interestingly, not only per capita uptake but also density itself of *H*. *filiformis* was significantly positively correlated to functional group diversity (Table 3). The negative (*M*. *liliana*) and positive (*H*. *filiformis*) effects of key-species density on per capita uptake in smaller surface-feeders was also mirrored when their density was considered as a response variable. For example *P*. *aucklandica* density was also negatively correlated with *M*. *liliana* density (Spearman ρ = -0.34, p < 0.05, S1 Table) whereas *Nucula* sp. and *P*. *aucklandica* densities were positively correlated to *H*. *filiformis* density (ρ = 0.50–0.75, p < 0.05, S1 Table). On a larger scale, these similarities between uptake and abundance could help explain why few spionids were found at the *M*. *liliana* dominated site. Thrush et al. [19] in a field experiment removed large *M*. *liliana* which resulted in increases in the density of *P*. *aucklandica* and *A*. *trifada*. In agreement with these findings Baltic Sea clam and amphipod abundances are negatively correlated in the field, and so are their uptake rates in laboratory experiments [61, 48]. Moreover, the negative relationship between meiofauna uptake rates and macrofaunal species diversity due to interference competition found in experimental work agree well with field data on meiofaunal abundance and biomass; both decreasing with higher macrofaunal diversity [36].

Although the spionid *P*. *aucklandica*, the amphipod *Parawaldeckia* sp. and the orbiniid *Naineris* sp. all had high per capita uptake and high densities, their small body mass (and hence low body nitrogen content), still down-weigh the importance of these species to total community uptake of macroalgal nitrogen. In contrast, *M*. *liliana*, which had a low per capita uptake during the experiment, most likely due to its slower growth and turnover relative to polychaetes and amphipods, nevertheless was the top or second most important species for total macroalgal nitrogen uptake in the community, when taking its large body size into account. The orbiniid *S*. *cylindrifer* had both highest per capita uptake and a large body size meaning that a large amount of *Ulva-*derived nitrogen was taken up in its tissues (Figs 2 and 3), suggesting it is a key-species for conversion of detritus to secondary production in this ecosystem. Due to competition, or other factors, the other orbiniid species had either too small body mass (*Naineris* sp.) or too low abundance and low per capita uptake (*O*. *papillosa*) to replace the function of *S*. *cylindrifer*. The fact that *S*. *cylindrifer* dominated macrofaunal community uptake suggests little redundancy for this particular ecosystem function during the initial rapid breakdown of macroalgal detritus. *M*. *liliana* and *H*. *filiformis* on the other hand, were the only representatives of their respective functional groups, meaning that species and functional identity cannot be differentiated, hence it is impossible to distinguish between the redundancy and rivet hypotheses. Indirectly, however, our results lend some support for the redundancy hypothesis, since functional group diversity (Shannons H’FG) contributed significantly in explaining both per capita uptake and total community uptake. As expected from the redundancy hypothesis, functional group diversity correlated positively with community uptake only in the *Macomona* site which had low numbers of species and functional groups (Fig 4A). This observation that an ecosystem process rate saturates at a rather low number of species has been shown from experimental work with synthetic assemblages representing e.g. soil communities, but is rarely shown in natural assemblages [2, 14, 62]. However, the relatively short duration of the experiment limits uptake of the labelled nitrogen by slow growing or predatory (or omnivorous) animals. It is likely that the importance of species richness for detrital uptake increase over larger spatial and temporal scales, as has been shown for ecosystem processes (e.g. biomass production and cover) in both terrestrial and aquatic systems [2, 63, 64].

*S*. *cylindrifer* had no effect on per capita uptake in other species (with the possible exception of *Parawaldeckia* sp.). *H*. *filiformis*, on the other hand, did not have a large uptake itself but instead facilitated uptake for surface-feeders through its unique bioturbation mode or by pre-conditioning the detritus into finer particles or more palatable material. The effect of *M*. *liliana*, also the only representative of its functional group (deposit-feeding large bivalves) was more ambivalent, since it negatively influenced per capita uptake of other, smaller surface-feeders through either exploitative and/or interference competition. However, as hypothesised it had a positive effect on *H*. *filiformis*, which in turn was positively associated to uptake rates of other community members. Finally, the largesize of *M*. *liliana* resulted in this species dominating community uptake of macroalgal nitrogen at the *Macomona* site, supporting the importance of large body size for ecosystem functioning [33, 36, 65].

In conclusion, our results demonstrate the importance of species identity, body size and density for ecosystem functioning, showing that large key-species determine uptake of algal detritus by macrofauna. These findings highlight the complex interactions underlying loss of ecological services and underscore the importance of understanding compositional and density changes of key-species with declining biodiversity.

## Supporting Information

S1 Fig Representative photographs of core surfaces 2 d after *Ulva* detritus addition illustrating inter-site differences in burial of the added material (green colour). (A) *Austrovenus* site showing *A*. *stuchburyi* and attached anemones not buried in the sediment. (B) *Macomona site* and (C) a core without large bivalves.
(TIFF)

S1 Table Species composition (number of individuals) for each core. (XLSX)

S2 Table Total biomass for each species for each core. (XLSX)

S3 Table Initial isotope values and C and N content of infauna from both sites. (XLSX)

S4 Table Isotope values and C and N content of infauna at the end of the experiment. (XLSX)
