# Supplementary figures and images for: Density of Key-Species Determines Efficiency of Macroalgae Detritus Uptake by Intertidal Benthic Communities
Source: PLoS One. 2016 Jul 14;11(7):e0158785. doi: 10.1371/journal.pone.0158785 (PMC4945087; doi:10.1371/journal.pone.0158785)

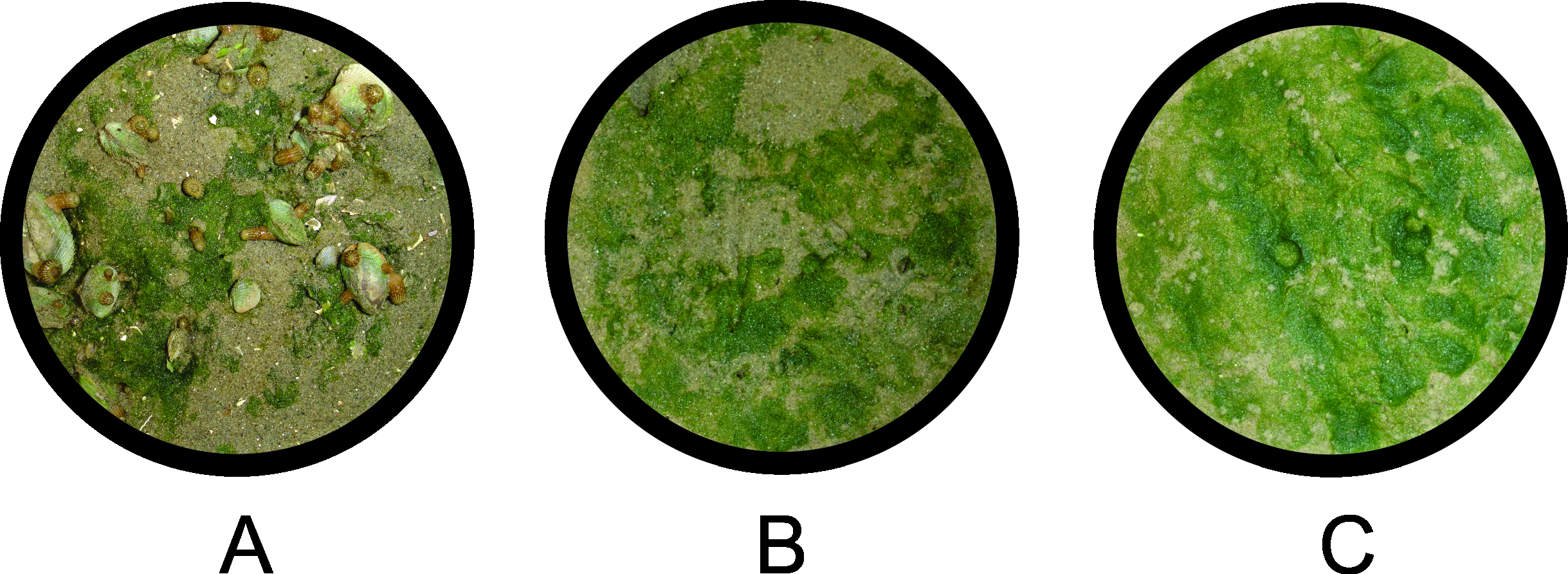

Supplement: S1 Fig — (A) Austrovenus site showing A. stuchburyi and attached anemones not buried in the sediment. (B) Macomona site and (C) a core without large bivalves. (TIFF) [file pone.0158785.s001.tiff]
